# In the mood of the other: emotional contagion, empathic knowledge, and intuitive diagnosis in psychiatry

**DOI:** 10.3389/fpsyg.2025.1676449

**Published:** 2025-12-19

**Authors:** Andrea Altobrando, Leonardo Zaninotto

**Affiliations:** 1 FISPPA Department, University of Padova, Padua, Italy; 2 Department of Mental Health, ULSS 6 Eugenea, Padua, Italy

**Keywords:** Max Scheler, Edith Stein, atmospheric diagnosis, diagnostic intuition, empathy, phenomenological psychiatry, emotional contagion

## Abstract

In this study we critically examine the phenomenological foundations of intuitive diagnosis in Psychiatry by integrating Max Scheler’s concept of emotional contagion with Edith Stein’s three-stage model of empathy. We argue that what Scheler calls emotional contagion offers a useful pre-reflective, bodily-affective resonance that precedes and facilitates deeper empathic understanding of the subject’s experience. Then, we suggest that Stein’s analysis of empathy, which is based on a three-step process—i.e., the emergence of the other’s experience, its imaginative explication, and the final comprehensive objectification—may account for the role of imaginative empathic immersion in diagnostic assessment. This imaginative engagement, grounded in bodily co-originality, allows clinicians to apprehend the subject’s world beyond mere perceptual awareness. We contend that Scheler’s emotional contagion and Stein’s model of empathy can be productively harnessed within a comprehensive diagnostic framework to provide a raw intuitive and imaginative substrate for further cognitive elaboration.

## 1 Introduction

Several ‘classical’ studies have shown that psychiatrists often make their diagnoses within the first few minutes of an interview (Cooper, 1983; Kendell, 1975; Sandifer et al., 1970), usually without any explicit awareness of the process (Gauron and Dickinson, 1966). Within phenomenological Psychiatry, intuitive diagnosis has been extensively explored (Jaspers, 1913; Minkowski, 1927; Rümke, 1941), being referred to by various terms, such as “atmospheric diagnosis” (Tellenbach, 1968), “diagnosis by intuition” (Wyrsch, 1946), “diagnosis by feeling” (Binswanger, 1924), “*diagnostique par pénétration*” (Minkowski, 1927), and “knowledge through the relationship” (Schneider, 1925). This phenomenon raises several important questions for psychiatric methodology: what role do the psychiatrist’s first impressions play in the diagnostic process? Are her ‘intuitions’, ‘feelings’, and ‘impressions’ reliable? And how do such elements affect the scientific foundations of Psychiatry as a medical discipline?

With the advent of modern technologies, Medicine has been overwhelmed by a climate of suspicion toward traditional clinical methods. Physicians began to distrust their own senses, and the classical tools of history-taking and direct physical examination progressively lost their importance.^1^ The physician’s subjectivity and its clinical validity were increasingly distrusted in favor of the supposed objectivity and reliability of technological devices. The same process involved Psychiatry, as well.^2^ In contrast to its early focus on the importance of a prolonged and direct relationship with the subject’s body, feelings, and thoughts,^3^ over the last decades, the “criteriological revolution” of the DSM (Fuchs, 2010a) has placed greater emphasis on quantitative, reproducible, and objectively measurable phenomena—at the expense of the qualitative, idiographic, and sensitive dimensions of the clinical encounter.

From the perspective of diagnostic manuals, diagnosis is expected to be made from a detached, third-person perspective, uniquely based on observable and reproducible behavioral phenomena, which are assumed to be independent of the context and the observer. Objectivity is supposed to be possible only by discarding the psychiatrist’s impressions and emotions and adopting what has been called a “point of view from nowhere” (Sholokhova, 2018).^4^ The clinician is not required to feel anything, and the “first-sight diagnosis”—something most psychiatrists report experiencing in daily practice—finds no place in the epistemology of manualized Psychiatry.

However, over the last decades, there has been an increasing interest in the role of intuitions in diagnostic reasoning and clinical decision making in Psychiatry (Srivastava and Grube, 2009; Fuchs, 2010b; Fernando et al., 2013; Morrison, 2014; Gupta et al., 2019). With few exceptions (Fuchs, 2010b; Nordgaard et al., 2013; Gupta et al., 2019), most theoretical models tend to reproduce a modified version of the medical approach, where diagnosis is conceived as an explicit, cognitive process, based on a third-person perspective, and aimed at categorizing the subject’s experience according to some pre-defined diagnostic criteria.

Other models are based on a second-person approach, which, by drawing on a phenomenological understanding of social cognition (Gallagher, 2001), refuses the Cartesian dualism of body and mind, and emphasizes the role of the pre-reflective intentional connection between bodies. According to this perspective the other’s emotions and intentions can be pre-reflectively grasped from the “expressive unity” of the body (Scheler, 1974), meant as an integrated psychophysical whole, in which gestures, attitudes, and expressions are immediate vehicles of emotions and intentions (Gallagher, 2001). Further, as it is conceived by the phenomenological perspective, the notion of empathy is based on a second-person, embodied process (Zahavi, 2010).

Within this framework, a considerable amount of research has been produced on the role of intersubjective phenomena in the diagnostic process in Psychiatry, exploring how the clinician’s emotions and intuitions may play a significant role in the assessment of mental disorders (for a comprehensive overview, see Biondi et al., 2022). A new psychometric instrument, named Assessment of Clinician’s Subjective Experience (ACSE) (Pallagrosi et al., 2014), has also been developed to assess the emotional, cognitive, and bodily experiences of the clinician during her interaction with subjects suffering from mental disorders, proving good psychometric properties, such as factorial validity and test re-test reliability (Pallagrosi et al., 2016; Picardi et al., 2021).

In this paper, we aim to show how certain concepts developed in the early phenomenological tradition^5^ can offer valuable tools for understanding the intuitive-emotional components of the diagnostic process in Psychiatry. We will specifically focus on Max Scheler’s notion of emotional contagion and Edith Stein’s three-step account of empathy.

In our view, Scheler’s (1974) concept of emotional contagion (*Gefühlsansteckung*) provides a valuable framework for understanding the initial “sensitive” dimension of psychiatric diagnosis, encompassing its intuitive, affective, unintentional and non-verbal aspects. This nascent and foundational moment of the clinical encounter may represent a brief window of time in which the “raw material” coming from the intersubjective space can manifest itself still unfiltered by the clinician’s “prejudices and assumptions” (Jaspers, 1913). Scheler’s emotional contagion is a pre-reflective, involuntary sharing of emotional experiences. Unlike other forms of social cognition, it does not require an explicit understanding the other’s experience.

We argue that emotional contagion may represent the “ground zero” of the diagnostic process—the fundamental moment of sharing an emotional experience, before any intentional movement is explicitly expressed, where this immediate, pre-reflective contact with the other’s experience simply “happens.” However, while this early phase provides a raw foundation for the diagnostic process, it is insufficient for a comprehensive understanding of the subject’s experience or for subsequent accurate therapeutic reflection. These are made possible only through a secondary and derivative component of intuitive diagnosis, going beyond emotional contagion yet building upon it, where the distance between self and other is established, and the experience can be thematized.

In our view, this component may be drawn from Edith Stein’s three-level analysis of empathy (Stein, 2008). According to Stein, empathy (*Einfühlung*) unfolds in three steps: (1) an immediate perception of the other’s thoughts, emotions and intentions through her expression; (2) an inner imaginative re-enactment of that experience within oneself; and (3) a comprehensive reflective understanding that recognizes it as the other’s own feeling. This process allows genuine intersubjective understanding while preserving the distinction between self and other. In this case, the subject’s experience, however distorted or alien it may appear, can become the object of an imaginative process of re-elaboration grounded in an empathic understanding which, while originating from a perceptual dimension, is enriched by imaginative and cognitive components (Zaninotto and Altobrando, 2022).

Our central claim is that a comprehensive account of the different stages of the diagnostic process must include both emotional contagion and multilevel empathy. Drawing on Scheler’s and Stein’s insights into intersubjective experience, we aim to clarify whether—and how—the direct, and at least partially pre-conceptual, relationship with the subject plays a crucial role in psychiatric diagnosis, especially in what is often referred to as diagnostic intuition. This pre-conceptual framework of the clinical encounter is subsequently elaborated through secondary cognitive and inferential processes, which serve to integrate and enrich the insights gained from affective resonance and the initial stages of empathic understanding. These processes incorporate content drawn from the subject’s self-narration, information from additional sources, and the clinician’s accumulated experience, theoretical knowledge, and overall personal sensitivity.^6^

Moreover, by engaging with the phenomenological tradition, we also aim to outline a kind of phenomenology of the clinical encounter, understood from the technical and specialized perspective of intuitive diagnosis. This includes identifying the specific conditions that make such an encounter possible and defining its methodological distinctiveness. Referring to Husserl, we aim to offer “a clear and rigorous elucidation of the knowledge and know-how that underlie effective Psychiatry” (Lanteri-Laura, 2007, p. 45).

## 2 Falling into an emotional state: emotional contagion (*Gefühlsansteckung*)

In phenomenological tradition, several analyses of intersubjective experience have been developed, many of which may offer valuable insights into the dynamics of the earliest phase of the clinical encounters. We believe Scheler’s (1974) concept of emotional contagion (*Gefühlsansteckung*), as outlined in “The Nature of Sympathy”, may help clarify a specific type of intersubjective experience that contributes to the very early stages of intuitive diagnosis. At the same time, this concept may shed light onto the reasons why direct, substantive, and “sensory” encounters with subjects—those that involve the clinician’s embodied self—can play a crucial role in psychiatric assessment.

Scheler observes that there are emotional experiences we undergo simply by being in the presence of others, even when those others are not the focus of our conscious attention. In emotional contagion, we are not explicitly attending to others or their emotional states; rather, we are moved by them, and come to partially feel like them, without consciously thematizing their experience. This process is unintentional and unconscious, and it primarily concerns the quality (the “how”)—rather than the object (the “what”) - of emotion. In emotional contagion we simply find ourselves in a certain emotional state—anxious, bored, angry—without being aware of having entered it. This is characteristic of moods and affective states in general, which often arise without intentional action or awareness. Furthermore, their objects are frequently unclear or absent. As Heidegger famously claimed, moods (*Stimmungen*) are essentially objectless (Heidegger, 1962).

What’s distinctive about emotional contagion is that, although we may be aware of and observing another person—even recognizing their mental state—the mood we “catch” is not the result of perceiving the other’s intentions as such. Rather, it arises non-thematically, before conscious interpretation. In Schelers’s words: “One enters a tavern where there is merriment and becomes merry oneself; children infect one another with their laughter; mourning-women with their whining; or the dreary rainy weather makes one oneself subdued.” (Scheler, 1974, p. 25).

We believe that something similar can happen at the very early stage of the diagnostic process, when the clinician happens to be immediately and pre-reflectively permeated by an emotional state. For example, it may happen that upon entering a room where someone is in a state of paranoid alertness, one immediately senses an atmosphere of tension and anxiety; or that, when faced with a person experiencing a severe depressive condition, one feels a sense of prostration and emotional numbness. These phenomena are supposed to come well before any explicit intentional movement is established and may represent the ground for what Messas et al. (2022) have called “affective resonance”, that is the first intersubjective evidence from which a phenomenologically oriented diagnosis is made. In this case, the clinician’s emotional response arises independently of any overt recognition of the subject’s emotional state, yet this spontaneous “feeling with” the subject may prove foundational to later diagnostic insight. In Minkowski’ s words: “we will feel *with* the patient, and this will be an important element of our psychiatric judgement” (Minkowski, 1927, p. 94).

Recalling Scheler’s aforementioned words, we hypothesize that emotional contagion represents one of the constitutive mechanisms of what, in a certain current of aesthetics, are referred to as “atmospheres” (Tellenbach, 1968; Schmitz, 1969a,b; Böhme, 1995, 1998; Andermann and Eberlein, 2011). “Atmospheres” are defined as affective-spatial fields that pervade environments and, in general, the inter-subjective space.^7^ They convey general moods (e.g., tension, warmth) and are sensed pre-reflectively, allowing subjects to “tune in” to the emotional tone of an environment or a relationship (Griffero, 2010). By translating the concepts, we might infer that emotional contagion represents one of the components of “atmospheric diagnosis,” as developed by Tellenbach (1968). In Tellenbach’s conceptualization, “an atmospheric element, though being not objectifiable […], still remains qualifiable, especially in those situations in which it can be perceived as a medium of intersubjectivity.” (pp. 55–56). As an example, Tellenbach suggests that an “atmospheric trace” may be experienced even in front of the mildest forms of melancholic depressions, as a specific sensation of withering or loss of freshness, of “*aviditas* instead of *viriditas*” (p. 62). Similarly, emotional contagion may originally convey that sense of detachment and relational “misalignment” that pervades the inter-subjective space in the “schizophrenic” contact, as described by the concept of “praecox feeling,” developed by the Dutch psychiatrist Rümke (1941). In Rümke’s conceptualization, the “schizophrenic coloration” of the encounter is detected even after a very brief mental state examination, where “it becomes clear to the psychiatrist that his empathy is lacking (and) it is impossible to establish contact with (the subject’s) personality as a whole” (p. 168). In this case we may argue that the “praecox feeling” can be understood as an inter-subjective phenomenon, arising from the patient’s own experience of detachment and empathic misalignment, which then resonates in the inter-subjective space and permeates the clinician’s perception as an experience of contagion (Vial et al., 2024).

A crucial feature that sets emotional contagion apart from empathy and sympathy is the absence of a reflective distinction between self and other. In empathy and sympathy, the other’s emotional state is a more or less explicit object of consciousness, and the boundary between self and other is maintained (Scheler, 1974).

In this regard, we should consider that Scheler’s broader account of intersubjectivity includes various other relational forms, some of which may also play important roles in psychiatric diagnosis, at least in its phenomenological conceptualization. The fundamental importance of emotional contagion for diagnosis can emerge more clearly when compared with some of these other forms of intersubjective experience. In *Zur Phänomenologie und Theorie der Sympathiegefühle* (Scheler, 1913), Scheler outlines a taxonomy of intersubjective experiences, where, besides emotional contagion, two further categories seem particularly relevant to clinical practice: “vicarious feeling” (*Nachfühlen*, sometimes translated as “perception of other subjects” or *Fremdwahrnehmung*), and “sympathy” or “fellow feeling” (*Mitgefühl*)—literally co-feeling.

Vicarious feeling differs from fellow feeling in that the former involves perceiving the other’s emotional state while maintaining distance—without participating in or emotionally responding to it. In contrast, fellow feeling involves sharing in the other’s emotion and adopting a certain evaluative stance toward it. For instance, one might feel joy at seeing a friend’s pleasure in eating cake—a second-order emotion distinct in both quality and content from the friend’s original pleasure. Although fellow feeling may be ethically desirable in clinical contexts, it is neither necessary nor always beneficial for diagnosis and treatment. In some situations, it could impair clinical objectivity. One might argue that while fellow feeling could motivate therapeutic action (e.g., helping a subject out of compassion), this motivation could equally stem from professional duty. Thus, fellow feeling does not provide decisive access to the subject’s mental state—it presupposes it, rather than revealing it. Clinical effectiveness does not depend on sympathy: clinicians can be unsympathetic yet highly competent in diagnosis (though this may not be true for treatment).^8^

Vicarious feeling, by contrast, seems more crucial to psychiatric diagnosis. The ability to directly perceive the subject’s mental state—without imaginative inference or emotional identification—is fundamental. This type of perception, according to Scheler, is immediate and non-analogical. To describe this phenomenon, he uses the term *Fremdwahrnehmung*, that is neither a judgment nor a simulation, but rather a straightforward perception: “For we certainly believe ourselves to be directly acquainted with another person’s joy in his laughter, with his sorrow and pain in his tears, with his shame in his blushing … and with the tenor of his thoughts in the sound of his words. […] If anyone tells me that this is not ‘perception’ […] I would beg him to turn aside from such questionable theories and address himself to the phenomenological facts.” (Scheler, 2008, p. 260).

Here, Scheler affirms a phenomenological approach to intersubjective understanding. Rather than starting from theories or ontological assumptions, one should begin with the experience itself and its intuitive structure.

Scheler argues that in our interactions with others, we immediately perceive their emotions through expressive features—gestures, voice, posture. Expressivity is the key to experiencing others as embodied beings (*Ausdruckseinheiten*). The living body (*Leib*) is not merely a physical object, but the primary medium through which the world—and others—are revealed. As Husserl also emphasized, the *Leib* is always already embedded in the world, and the world is given to us in and through embodied perception (Husserl, 1952). In this sense, other-perception always involves what Cassirer called expression-perception (*Ausdruckswahrnehmung*): understanding another’s emotions through their expressive embodiment, without necessarily reproducing those emotions in oneself [Cassirer and Bollingen Foundation Collection (Library of Congress), 1953].

Vicarious feeling certainly constitutes a form of understanding highly relevant to clinical practice. It apparently allows for a kind of “immediate” awareness of the subject’s mental states—an understanding that does not require feeling the same emotion. Scheler gives striking examples: a torturer, for instance, may be skilled at discerning a victim’s pain and eliciting further suffering, even without sharing that suffering. Likewise, a comedian or clown might masterfully read and shape the audience’s emotions without participating in them. In Psychiatry, the clinician likewise needs to grasp the subject’s emotional state—often pre-reflectively and perceptually—without necessarily sharing it. For example, a psychiatrist in front of a severely depressed subject may vicariously feel his sense of emotional emptiness and despair, even when it is not verbally expressed, and without experiencing it herself. If this is how matters stand, should not we believe that vicarious feeling, rather than emotional contagion, is the fundamental basis of psychiatric diagnosis, at least in its phenomenological conceptualization?

## 3 Emotional contagion or vicarious feeling?

Based on Scheler’s account of vicarious feeling, one might reasonably argue that, in psychiatric practice, the clinician’s task is not primarily to share the subject’s emotions and sensations—as it occurs in emotional contagion—but rather to understand them and help the subject overcome her suffering. This capacity is more appropriately associated with vicarious feeling. Sympathy, although it may play some role in the diagnostic process, does not appear to be necessary, and could even be counterproductive.

Following this line of reasoning, we may conclude that the clinician needs only to be capable of other-perception—that is, vicarious feeling—and that no further intersubjective involvement is required. Even if what Scheler calls emotional contagion can occur, it might be argued that the clinician should either avoid it entirely, or at the very least be able to quickly disengage from it and regain a critical, perceptual distance from the subject and her emotional state. Clinical diagnosis, after all, requires a sober and lucid view of the subject’s experience—not a direct emotional involvement in the suffering itself.

In emotional contagion, we do not perceive the other’s emotions from a distance, but rather we feel them within ourselves—or rather, feel ourselves “within” them. As Scheler himself warns, emotional contagion can be a potentially dangerous state of mind. In extreme cases, it may even result in a loss of awareness of one’s own distinction from others. One is simply carried away, beyond or beneath the level of self-control and self-conscious differentiation from others (Scheler, 2008). At its most extreme, emotional contagion might resemble being swept up in a euphoric mass state.^9^

If that is the case, then one might reasonably conclude that—even if emotional contagion is possible—it is not at all beneficial, and that the clinician’s responsibility is precisely to avoid such contagion, not to cultivate a susceptibility to it.

However, we believe this conclusion is only partially correct. There is no question that a “sick” clinician is neither helpful nor desirable. That is certainly not what we advocate as appropriate—let alone necessary. Still, the clinician must find a way to access the experiential material upon which her diagnostic activity depends. In this respect, and only in this respect, we argue that something akin to emotional contagion can be diagnostically valuable.

This does not mean that the clinician should linger in the emotional state of the subject or surrender to it. While we fully acknowledge the potential risks of emotional contagion and the need to regulate it, we maintain that the phenomenon it describes can be productively employed in the context of a fully embodied and comprehensive psychopathological diagnosis.

There is no doubt that vicarious feeling is essential for psychopathological diagnosis. The clinician must be capable of perceiving the subject’s mental life without falling into the same affective state. And in many, if not most, cases, a diagnosis can be successfully made based on vicarious feeling alone. Still, we argue that emotional contagion might assist in initiating a more nuanced and complete diagnostic process—and may even be, at least implicitly, a necessary aspect of a phenomenologically oriented diagnosis.

In short, we emphasize the possible utility of emotional contagion for two main reasons: first, because it appears to capture some core elements of the clinician’s experience within “atmospheric diagnosis” (see above); second, because it seems to mark the moment in the inter-subjective encounter when the “raw” diagnostic material can be most purely and directly perceived.

To clarify this idea, a more careful examination of the layers within the inter-subjective relationship is needed—particularly of what we refer to as other-perception, or empathy. In this regard, Edith Stein’s phenomenological analysis of empathy provides a clearer account of its layered structure. Compared to Scheler’s distinction between vicarious and fellow feeling, Stein offers a model that enables us to identify more precisely the role that emotional contagion may play.

## 4 From other-perception to imagining oneself in the other’s emotions and intentions

Scheler himself acknowledges that in vicarious feeling one almost feels in oneself the other’s emotions, feelings, and intentions. That is why Scheler uses the word *Nachfühlen*, which could literally be translated as “after-feeling,” and implies a kind of repetition, or resonance, of the other’s feeling in oneself. Scheler decisively denies that this amounts to any kind of analogical understanding or simulation. Although Edith Stein also denies that other-perception is based on some kind of analogical inference, she shows how some kind of virtual simulation in the sense of an imaginative repetition is indeed part of the process through which we understand others. In this regard, Stein’s analyses fruitfully integrate Scheler’s concepts of emotional contagion and vicarious feeling. Stein’s analyses allow us to understand the role of imagination in empathy, a role that is underestimated, indeed, and, to a certain extent, even ruled out by Scheler. As Svanaeus (2017) has very well shown, Stein proposes an understanding of empathy as a three-step process, where imagination plays a fundamental role for the specifically cognitive experience of the other. Stein’s analysis, indeed, allows us to understand empathy as a complex phenomenon that should neither be reduced to the direct perception of the other’s emotions, feelings, and sensations, nor to a sympathetic posture towards the other’s experience. As we have seen, in Stein’s conceptualization empathy is regarded as a three-step process where: (1) the experience of the other emerges as a meaningful complex (the emergence of the experience) and becomes the object of (2) an explication through an imaginative account (the fulfilling explication), in order to (3) return to a more comprehensive understanding of the experience (the comprehensive objectification) (Svanaeus, 2017).

Let us, then, see how Stein herself describes the three-steps process:

“Now to empathy itself. Here, too, we are dealing with an act that is original in the sense of being a present experience but non-original as regards its content. And this content is an experience that can, again, come in many different forms, as memory, expectation, or imagination. When it suddenly appears before me it faces me as an object (for instance, the sadness I “read” in the other’s face). But when I inquire into its implied tendencies (when I try to bring the other’s mood to clear givenness to myself), the experience is no longer an object for me but has pulled myself into it. I am now no longer turned towards the experience, but instead I am turned towards the object of the experience. I am at the subject of the original experience, at the subject’s place, and only after having fulfilled a clarification of the experience does it appear to me as an object again. […] Consequently we have in all considered cases when experiences [of other subjects] are appearing to us three stages or modalities of accomplishment, even though in each concrete case not all of the three stages are accomplished, but we often settle with stage one or two: 1. the emergence of the experience, 2. the fulfilling explication, and 3. The comprehensive objectification of the explicated experience.” (Stein, 2008, pp. 18–19).

For Stein, it is not the case that anytime we become aware of another subject’s feelings, emotions, and intentions, we start a full empathic process. We may sometimes perceive another’s experience yet choose not to focus on it beyond that initial awareness. Therefore, we would agree with Svanaeus (2017), who claims that “[r]ather than taking empathy to be a basic, obligatory ingredient in all everyday encounters in which we perceive and act with others, it is (…) more enlightening to comprehend (it) as an attempt to understand the experiences of the other person in their own right.” (p. 178).

Svenaeus’ understanding of Stein, being explicitly directed towards an elaboration of empathy that can be useful for medical praxis, is particularly appropriate to also understand the specific case of psychopathological praxis. In this regard, Svanaeus (2017) poignantly shows that Stein’s understanding of empathy cannot be reduced to the mere direct perception of the other’s feeling.^10^ The empathic experience does not merely consist in the awareness of the other’s experience, but it is a process during which the empathizer makes something of this awareness and its content. Moreover, the awareness itself is motivated, i.e., it raises for some practical reasons within one’s perceptual field. In the case we are discussing here, the need-interest corresponds to the clinical endeavour of the psychiatrist: what we could call her “attuned interest” (Svanaeus, 2015, 2017) for the subject’s experience of suffering and alienation. In other words, the imaginative process of the clinician is triggered by her interest in understanding the subject’s inner world (Magrì, 2015).

As an example, a psychiatrist meets a subject experiencing auditory hallucinations and paranoid delusions. After entering the room and immediately sensing an atmosphere of tension and anxiety (emotional contagion), the subject’s suspicious glances, tense posture, and guarded speech allow the psychiatrist to perceive (emergence) her inner experiences of fear and suspicion as a meaningful complex (vicarious feeling). Guided by an attuned interest in the patient’s well-being, the clinician then imaginatively engages with these expressions of fear, mistrust, and hypervigilance to form a comprehensive, *gestaltic* view of the subject’s paranoid experience (fulfilling explication). Finally, she returns to a more comprehensive understanding of the experience, grounded not only in clinical knowledge and phenomenological insight but also in the patient’s narrative, leading to a nuanced, empathically informed understanding of the subject’s paranoid delusional state (comprehensive objectification).

Furthermore, though this is not the focus of our argument, we should consider that the clinician may also experience what others may feel in front of the suffering subject, for example the helplessness family members feel in response to their related’s repeated suicidal behaviors. This is a mediated, second-order empathy with third-party reactions, and not the subject’s affect (Stein, 2008). In clinical terms: when a borderline patient enacts suicidal behavior, the therapist may feel the same helplessness reported by the patient’s spouse—not via contagion of the patient’s affect, but via mediated empathy with the family member’s lived response. This second-order empathy reveals the interpersonal field the patient generates.

Further, we believe that “affective resonance” (Messas et al., 2022) and the three steps of empathy are structured in a unidirectional foundational relationship: step one presupposes affective attunement, step two presupposes step one, and step three presupposes step two, but not vice versa (Husserl, 2001; Rota, 1989). This foundational structure does not require any strict temporal succession; the steps may occur simultaneously as a unified act. Importantly, higher-level processes can influence lower levels. For example, the imaginative phase may shape the underlying emotional contagion, and the objectifying step may modulate perception of bodily expressiveness. Conversely, emotional contagion may directly impact higher-level processing through the clinician’s emotional response. Rather than blurring the distinction between stages, such retroactive interactions highlight the importance of careful attention to all phase-including emotional ones-during clinical encounters and underscore the need for extensive training to manage factors contributing to diagnosis.

## 5 Empathy and the body

Mere perception of the other does not allow one to truly enter and explore their emotional life—to follow where their feelings lead or what other mental states they might be linked to. Just as we cannot see what another sees without taking their perspective, we cannot understand the meaning of another’s emotions without imaginatively entering them.

Edith Stein’s analysis helps clarify the role of the perceiver’s body in empathy and its connection to imaginative activity. She also emphasizes a kind of sensory relationship at the root of empathy: the other’s emotions always manifest through their body. Yet this is not a matter of analogy—of recognizing expressions because they resemble our own. Instead, Stein, following Scheler, argues that the other’s body is immediately expressive of emotional life.

What is particularly relevant here is Stein’s claim that when we perceive a living body, we perceive feelings and intentions as emanating from it (Stein, 2008), enabling a form of co-original emotional experience. “Co-originality,” a concept shared with Husserl (1950), refers to an experience given directly (in the flesh), as opposed to indirectly or representationally. While the other’s body is perceived originally, their inner experiences cannot be—otherwise, we would experience them as our own. Empathy thus shares features with perception (in its immediacy) and imagination (in the indirect nature of its content).

According to Stein, then, perceiving the other cannot be separated from co-experiencing their emotions. This quasi-feeling is accompanied by an inclination to imaginatively immerse oneself in the other’s mood. As she writes, “when I inquire into its implied tendencies […] the experience is no longer an object for me but has pulled myself into it” (Stein, 2008, p. 18). This moment is crucial: empathy draws us into an imaginative engagement, motivated by a desire (an “attuned interest”) to understand the other’s emotional world.

Importantly, this imaginative immersion differs from both Scheler’s vicarious feeling, which lacks real participation, and his notion of emotional contagion, where one is passively overtaken. In Stein’s account, the empathic pull is both imaginative and intentional—it arises from a cognitive and affective interest in the other.

Finally, the ability to imaginatively vary the emotional state initially perceived in the other enables one to explore it from within and, through what Husserl (1973) terms “eidetic variation,” to apprehend the essential structure of the experience it embodies. This phase—crucial for clinical diagnosis—marks the culmination of the empathic process. Although a detailed discussion of imagination’s role in psychiatric understanding lies beyond the scope of this paper, it should be emphasized that imagination is not merely an auxiliary faculty but a central component of our cognitive access to the other.

Furthermore, as we previously discussed (Zaninotto and Altobrando, 2022), we believe that this account of empathic understanding—one that involves a specifically imaginative component—may help to at least partially overcome the challenge posed by ‘un-understandable’ psychopathological experiences, such as ‘primary’ schizophrenic delusions and other.

## 6 Conclusion

It may appear that Stein’s three step model of empathy not only complements Max Scheler’s notion of emotional contagion but could potentially replace it—at least within the framework of a phenomenologically oriented framework. Stein’s theory clarifies both the way clinicians access a subject’s emotional world and how they move from direct perception to an imaginative engagement with her experience—an essential step in evaluating the nature of the subject’s mental condition. Moreover, Stein’s theory also seems to align with Tellenbach’s idea of “atmospheric diagnosis” (1968), where diagnostic intuition stems from the clinician’s bodily sensitivity and accumulated experience. In these situations, the clinician makes a largely passive, associative inference, reaching a diagnostic impression almost intuitively. From this view, Stein’s model seems sufficient to explain much of what constitutes intuitive clinical insight.

However, we must not overlook the distinctive value of emotional contagion. Unlike Stein’s conception of empathy, emotional contagion does not originate from a deliberate cognitive engagement. Rather, it is something the clinician undergoes—often preceding or occurring outside conscious intention. It emerges within the clinician’s subjectivity at a pre-conceptual and pre-intentional level, prior to any act of categorization and attuned interest.

In contagion, one does not merely imagine the other’s state—one feels it directly, even before identifying or interpreting it. Emotional contagion represents something that only a personal, embodied encounter between clinician and subject can provide. There must be a moment in which a resonance between the two occurs, where it is possible a kind of raw, pre-reflective access to the other’s affective life. This experience can serve as a “purer” starting point for further empathic and cognitive elaboration, precisely because it has not yet been shaped by the clinician’s prior assumptions or expectations. In fact, emotional contagion may help address a structural gap in Stein’s model. Her three-step process moves from perceiving the other’s emotions and intentions to imaginatively entering them. But the transition from a third-person observation to a first-person engagement—especially into an imaginary first-person perspective—requires an affective bridge. We hypothesize that what makes such a transition possible is precisely the kind of pre-reflective contagion described by Scheler. Without this initial affective pull, the shift from mere perception to imaginative immersion lacks a foundation. Thus, emotional contagion appears as a necessary complement to Stein’s theory for three main reasons:

1. While Stein emphasizes imagination as a secondary phase in the empathic process, genuine emotional contact often involves a more immediate, bodily-affective resonance. Emotional contagion may vary in intensity and duration, and the clinician must learn-through experience-when to allow or when to restrain it. While modern psychiatric training often stresses suppression, we propose that more nuanced training would teach clinicians how and when emotional contagion can serve a therapeutic purpose. That said, as we repeatedly underlined, emotional contagion alone is not sufficient for diagnosis. Without cognitive distance, the clinician risks becoming overwhelmed. Clinical insight requires a movement from lived affective resonance to imaginative exploration-from being emotionally ‘in touch’, or even ‘in continuity’ with the subject to forming a structured, diagnostic understanding. Emotional contagion can support this transition by providing a first-person anchor. Yet imaginative variation and typological categorization remain indispensable for arriving at a provisional diagnostic conclusion.
2. Emotional contagion allows access to the subject’s inner life outside the constraints of predefined diagnostic categories. This is crucial for avoiding premature or reductive classification. Rather than forcing the subject into a rigid taxonomy, the clinician can remain open to the uniqueness of the individual’s experience.
3. Ultimately, emotional contagion grounds the diagnostic process in the concrete, personal dimension of the clinical encounter. It is this bodily resonance that energizes the empathic and diagnostic work that follows. Being affected by the subject is not in itself sufficient for diagnosis, yet it remains an indispensable condition for it. Emotional contagion provides the raw epistemic ground of clinical practice, generating the sensory and affective inputs which, through the interpretive work of empathic imagination and clinical reasoning, enable an account of the patient’s condition while preserving their individual specificity.

## Footnotes

1 The main reasons for this powerful trend may be either a sense of discomfort with uncertainty or the fear of subjective observations. See Jauhar (2006).

2 From the genesis of Psychiatry as a medical specialty in the middle of the Modern Age up to the present day, the medical model has permeated the concept of mental illness (Black, 2005), laying the basis for the epistemic foundations of Psychiatry as a science, and giving the psychiatrist both a method for studying the nature and development of mental disorders, and a theoretical framework for her daily practice (Andreasen, 2007). The model of medical semeiotics and clinical methodology has been applied to the examination of the mentally ill, where the collection of (behavioral) signs and (experienced) symptoms could only be made by means of a direct “sensory” contact with the subject.

3 In the Introduction to the first edition of his *Traité*, Philippe Pinel wrote: “*The habit of living constantly among the insane, of studying their habits, their different characters, the objects of their pleasures or their repugnance, the advantage of following the course of their alienation during the day, the night, and the different seasons of the year. all of this must necessarily communicate to the intelligent and zealous man a large amount of knowledge and a bunch of minute details that cannot be collected by those doctors, usually having a narrow view, that limit themselves to short visits*.” (Pinel, 1800, p. XLV).

4 Although statistical manuals have been regarded as “a significant reaffirmation of (the) medical identity of Psychiatry” (Klerman et al., 1984), they are a long way from the traditional psychopathological assessment based on clinical examination. Under the guise of objectivity, they not only risk underestimating the contribution of the clinician’s experience to the diagnostic process, but they also risk evading the problem of individuality and meaning, by considering the subject as a representative of a diagnostic category, and by excluding any contribution of subjectivity to clinical examination. They usually put accent on the single clinical fragment, which must be decontextualized to address the issue of reliability (Wakefield, 2010), and they are based on targeted questions, designed to find specific clusters of symptoms to establish a diagnostic entity.

5 When compared to the objectifying, morbus-oriented, biomedical model, the person-oriented tradition of psychopathology (Kraus, 1994) implies a sort of epistemological and methodological turn by the clinician, who is required to get involved into a close and unmediated relationship with the subject (Fuchs, 2010b). This may be the reason why skillful psychopathological assessment, perceived as challenging and time-consuming, is no more part of the core curriculum of mental health professionals (Parnas et al., 2013; Stanghellini, 2009; Andreasen, 2007) leaving most trainees in Psychiatry unsatisfied with the training they have received in psychopathology (Fiorillo et al., 2016).

6 Our integration of Scheler’s concept of emotional contagion with Stein’s three-step model of empathy may evoke Kahneman’s Dual-Process Theory (Kahneman, 2011), which distinguishes between fast, intuitive, affect-laden processing (Type 1) and slow, reflective, hypothesis-testing reasoning (Type 2). While this dual-process framework has been widely applied to clinical diagnosis (Norman, 2009; Croskerry et al., 2014), we see important distinctions from our own theoretical approach. The key difference lies in the role of the initial, intuitive stage. In Kahneman’s model, Type 1 processing—that is supposed to parallel emotional contagion—is considered capable of producing a diagnosis. In contrast, we argue that a proper clinical diagnosis can only emerge after the full engagement of Stein’s three-step empathic process. Emotional contagion alone cannot generate a diagnosis; rather, it triggers the clinician’s response, which may lead to the implementation of the empathic process. The two frameworks could potentially be integrated, with our empathic model providing a deeper, stepwise process for translating initial emotional resonance into diagnostic insight.

7 Although we find some proximity between the concept of “atmospheres” developed in the past decades on the basis of Tellenbach’s and Schmitz’s ideas, we need to point out that here we remain neutral as regard the ontological status of both atmospheres and the “stuff” that emotional contagion is made of. In emotional contagion, one partakes in a shared, diffused feeling—but this does not require positing a literal “emotional substance” transferred between individuals. We can remain within an individualistic ontology, where experiences belong to individuals and cannot be detached or transmitted like objects. Emotional contagion, then, should not be understood as the spread of emotion like a virus. Following Scheler’s insights—while avoiding ontological claims about collective or multi-subject feelings—we can instead view these situations as cases where one is immersed in a group behaving in a coordinated way. By enacting similar behavior, the individual comes to feel as others do. In this view, imitation may underlie the experience of feeling with and like others (Tomasello et al., 1993). This kind of experience deserves further investigation, particularly regarding the quasi-automatic, pre-reflective imitation studied under terms like “off-line imagination” or “off-line simulation” (Currie and Ravenscroft, 2002) and sometimes associated with mirror neurons theory (Gallese, 2007). While we cannot explore here the spontaneous, evolutionary basis, or pre-conscious nature of such mechanisms, we can tentatively understand emotional contagion as a form of off-line simulation—without assuming that emotions literally transfer between bodies. Whether emotions might be virologically conceived, or whether atmospheres have ontological status as “airy substances,” are questions best left for another discussion. While we lean toward a more traditional, individualist ontology, our focus here is on offering phenomenological descriptions—particularly of “atmospheric diagnosis” and the clinician’s experience—leaving deeper ontological commitments to future inquiry. As for a recent “analytic” proposal concerning the metaphysical status of atmospheres that does not commit itself to some kind of over-individual substance, see: Fernandez Velasco and Niikawa (2025).

8 For instance, even if a clinician strongly dislikes a subject, she might be still able and willing to work for the subject’s recovery because of their commitment to medical deontology.

9 However, emotional contagion should not be confused with what Scheler calls “identification” (*Einsfühlung*). Identification involves merging oneself with another person’s identity, whereas in emotional contagion there is no such personal merging—rather, one is swept up into a shared emotional state. Both may involve a loss of individual identity, but in identification, the self dissolves into another specific person, while in emotional contagion it dissolves into a collective state or an indistinct “we,” or perhaps even into a general loss of self.

10 Zahavi (2011), drawing on his reading of Husserl, argues that empathy is best understood as a perceptual experience: we do not need to *feel like* the other to recognize that they feel something. Whether this interpretation of Husserl is accurate is not our concern here. What matters is that Zahavi offers a compelling case for distinguishing empathic perception from emotional identification. Just as seeing something does not require being it, empathizing does not necessarily entail sharing the other’s experience. However, as Dullstein (2013) points out, this account is too narrow. It overlooks key insights from recent simulation theories of empathy, which emphasize empathy as an active, pragmatic, and goal-directed process [see, e.g., de Vignemont and Jacob (2012)]. In contrast to Zahavi’s model, these approaches stress the dynamic, participatory nature of empathic engagement. The debate between Zahavi’s and the Svenaeus–Stein views may ultimately hinge on terminology, since both acknowledge a basic level of experience in which the other’s feelings are registered without full emotional resonance or cognitive elaboration. This “*minimal empathy*” may indeed account for many ordinary encounters with others. Yet even at this basic level, we should not overlook the implicit meaningfulness of perceiving the other’s experience. The mere fact that another’s feeling enters our experiential field suggests some degree of relevance or salience to us. Whether this low-level awareness involves off-line simulation, *à la* Currie and Ravenscroft (2002), or mirror neurons activation, *à la* Gallese (2001), remains an open question. And we can also find naturalistic, evolutionary accounts—such as that suggested by Tomasello et al. (1993)—that explain why recognizing others’ emotions has survival value. What matters for our purposes is the next phase in Stein’s conception of empathy: when the other’s experience is no longer simply observed but becomes the very context within which I imaginatively or simulative move and situate myself. At this stage, empathy involves an imaginative act by the empathizer, making such imaginative experience essential to truly grasping another’s emotional life. If this is right, then Zahavi’s perceptual account is inadequate—particularly in clinical contexts. Empathy in therapeutic practice must involve more than detached observation; it requires at least a potential ethical or practical orientation towards the subject’s well-being.
